# The effects of phosphodiesterase-5 inhibitor sildenafil against post-resuscitation myocardial and intestinal microcirculatory dysfunction by attenuating apoptosis and regulating microRNAs expression: essential role of nitric oxide syntheses signaling

**DOI:** 10.1186/s12967-015-0550-9

**Published:** 2015-06-04

**Authors:** Qian Zhang, Guoxing Wang, Wei Yuan, Junyuan Wu, Miaomiao Wang, ChunSheng Li

**Affiliations:** Department of Emergency Medicine, Beijing Chao-yang Hospital, Capital Medical University, Beijing, 100020 China; Department of Emergency Medicine, Beijing You-yi Hospital, Capital Medical University, Beijing, 100050 China

**Keywords:** Sildenafil, iNOS, eNOS, Post-resuscitation myocardial dysfunction, MicroRNAs, Apoptosis

## Abstract

**Background:**

Recent experimental and clinical studies have indicated the cardioprotective role of sildenafil during ischemia/reperfusion (I/R) injury. Sildenafil has been shown to attenuate postresuscitation myocardial dysfunction in piget models of ventricular fibrillation. This study was designed to investigate if administration of sildenafil will attenuate post-resuscitation myocardial dysfunction by attenuating apoptosis and regulating miRNA expressions, furthermore, ameliorating the severity of post-microcirculatory dysfunction.

**Methods:**

Twenty-four male pigs (weighing 30 ± 2 kg) were randomly divided into groups, sildenafil pretreatment (n = 8), saline (n = 8) and sham operation (sham, n = 8). Sildenafil pretreatment consisted of 0.5 mg/kg sildenafil, administered once intraperitoneally 30 min prior to ventricular fibrillation (VF). Eight minutes of untreated VF was followed by defibrillation in anesthetized, closed-chest pigs. Hemodynamic status and blood samples were obtained at 0 min, 0.5, 1, 2, 4 and 6 h after return of spontaneous circulation (ROSC). Surviving pigs were euthanatized at 24 h after ROSC, and hearts were removed for analysis by electron microscopy, western blotting, quantitative real-time polymerase chain reaction (PCR), and terminal deoxynucleotidyl transferase-mediated dUTP nick end labeling (TUNEL) assay. Intestinal microcirculatory blood flow was visualized by a sidestream dark-field imaging device at baseline and 0.5, 1, 2, 4, and 6 h after ROSC.

**Results:**

Compared with the saline group, the sildenafil group had a higher 24-hour survival (7/8 versus 3/8 survivors, p < 0.05) and a better outcome in hemodynamic parameters. The protective effect of sildenafil also correlated with reduced cardiomyocyte apoptosis, as evidenced by reduced TUNEL-positive cells, increased anti-apoptotic Bcl-2/Bax ratio and inhibited caspase-3 activity in myocardium. Additionally, sildenafil treatment inhibited the increases in the microRNA-1 levels and alleviated the decreases in the microRNA-133a levels which negatively regulates pro-apoptotic genes. At 6 h after ROSC, post-resuscitation perfused vessel density and microcirculatory flow index were significantly lower in the saline group than in the sildenafil group.

**Conclusions:**

The major findings of this study are as follows: (1) sildenafil improved post-resuscitation perfusion of the heart, and thus reduced cardiac myocyte apoptosis and improved cardiac function; (2) sildenafil treatment inhibited the increases in the microRNA-1 levels, but alleviated the decreases in the microRNA-133a levels.

## Background

In the United States and Europe approximately one million patients suffer from out-of-hospital cardiac arrest (CA) [1]. Although initially a restoration of spontaneous circulation (ROSC) can be achieved in 25–50%, only 2–10% survive without major neurological deficit. Main reason for this poor outcome is the so called post-resuscitation syndrome [1]. Postresuscitation myocardial dysfunction, an important component of the “post-cardiac arrest syndrome”, is caused by ischemia/reperfusion (I/R) injury and includes primary manifestations such as arrhythmias, myocyte apoptosis, and contractile dysfunction [2]. Sildenafil citrate inhibits phosphodiesterase-5 (PDE-5), which catalyzes the breakdown of 39,59-cyclic guanosine monophosphate (cGMP), one of the primary factors involved in smooth muscle relaxation. Sildenafil induces the upregulation of endothelial NOS (eNOS) and inducible NOS (iNOS), which generate nitrogen oxide (NO) [3]. It is well established that sildenafil provided direct cardioprotection against ischemia and reduced apoptosis in cardiomyocytes subjected to simulated ischemia and reoxygenation through NO-dependent pathway [4]. We have previously demonstrated that, in a porcine model of CA, sildenafil improves the success of initial resuscitation and ameliorates myocardial dysfunction through improving myocardial metabolism [5]. Other investigators have also reported similar attenuation of myocardial dysfunction after ischemia in rats [6].

Individual microRNA (miRNA) is functionally important as a transcription factor because it has the ability to regulate the expression of multiple genes through binding to its target with imperfect or perfect complement [7]. In heart, miRNAs have been involved in several clinical scenarios, such as I/R injury and heart failure suggesting that regulation of their function could be used as a novel cardio-protective strategy [8]. In particular, miRNA-1 and miRNA-133a have been shown to be regulated after myocardium I/R injury [9]. More recently, miRNA-1 and miRNA-133a were shown to produce opposing effects on oxidative stress-with miRNA-1 being pro-apoptotic and miRNA-133 being anti-apoptotic [10]. However, the significance of miRNA expression and possible roles of miRNAs in post-resuscitation myocardial dysfunction are not well studied [11].

Changes in macrocirculatory hemodynamics and gas exchange during cardiopulmonary resuscitation (CPR) have been investigated extensively as predictors of outcomes in the restoration of cardiac function [12]. Yet the “microcirculation” and more specifically, the capillary exchange bed is likely to be the ultimate determinant of circulatory function. Current evidence indicated that microcirculation regulates tissue blood flow and is therefore more important than macrocirculation in tissue oxygen delivery and utilization [13]. Microcirculatory changes differ extensively among organs especially during the extreme low-flow state of CA and especially CPR.

Based on this background, the present study was designed to test the hypothesis that sildenafil decreases post-resuscitation myocardial dysfunction by enhancing the activation of iNOS and eNOS production and, acts on myocardial ischaemia-associated miRNAs and their functional significance in the modulation of cardiomyocyte apoptosis. Finally, this study was to investigate if sildenafil will ameliorate the severity of post-resuscitation microcirculatory dysfunction.

## Methods

### Ethics statement

This study was carried out in strict accordance with the guideline for animal care and use established by the Capital Medical University Animal Care and Use Committee. The study’s experimental protocol was approved by the Committee on the Ethics of Animal Experiments of Capital Medical University (Permit Number: 2010-D-013). Animals used in this study were handled in compliance with the Guiding Principles for the Care and Use of Animals expressed in the Declaration of Helsinki [14]. All animals were maintained in a specific pathogen-free environment in our facility, and were fed with standard chow and had free access to water. All surgery was performed under anesthesia and analgesia, and all efforts were made to minimize suffering.

### Animal preparation

Twenty-four inbred male Wu-zhi-shan miniature pigs aged 11–13 months with an average weight of 30 ± 2 kg were used in each part of this study [15]. The piglets were randomly assigned into three groups, sildenafil group (n = 8), saline (SA group, n = 8); and sham operation group (sham group, n = 8). Sildenafil was obtained from a 25-mg Viagra (Pfizer Australia) tablet that was dissolved in 50 mL saline, filtered and stored at 4°C. This solution was given once intraperitoneally in the dose of 0.5 mg/kg 30 min prior to VF [5]. The drugs were delivered in a randomized manner by the sealed envelope method. The vehicle (0.9% NaCl) was administered in the same manner and volume. After premedication with 0.5 mg/kg intramuscular midazolam, the animal was anesthetized by ear vein injection of propofol (1.0 mg/kg) and maintained in a surgical plane of anesthesia with intravenous infusion of sodium pentobarbital (8 mg/kg/h). All animals were intubated by a cuffed 6.5-mm endotracheal tube and ventilated by a volume-controlled ventilator (Servo 900C; Siemens, Munich, Germany) using a tidal volume of 8 mL/kg and a respiratory frequency of 12 breaths/min with room air. End-tidal CO_2_ was measured by an inline infrared cacographic (CO_2_SMO plus monitor; Respirometric Inc, Murrysville). Respiratory frequency was adjusted to maintain end-tidal CO_2_ between 35 and 40 mmHg before CA was induced. Room temperature was adjusted to 26 C, and body temperature was maintained at 37°C under an infrared lamp, and all efforts were made to minimize suffering. Fluid losses were compensated by an infusion of 30 mL/kg acetated Ringer’s solution during the first hour of preparation, followed by a continuous infusion of 2.5% glucose-electrolytes solution 8 mL/kg/h and acetated Ringer’s solution 20 mL/kg/h. All investigators performing CPR and interpreting the outcome assessments were blinded to the medication.

An angiographic catheter was inserted from the femoral artery into the aortic arch for collecting blood samples and for measuring aortic pressure. A Swan-Ganz catheter (7 Fr; Edwards Life Sciences, Irvine, CA) was advanced from the right femoral vein and flow-directed into the pulmonary artery for measurement of right atrial pressure, mean pulmonary arterial pressure (MPAP) and cardiac output (CO). The electrocardiogram and all hemodynamic parameters were monitored with a patient monitoring system (M1165; Hewlett-Packard, Palo Alto, CA). Animals with self-adhesive defibrillation electrodes located on the chest wall. A laparotomy was performed to expose the peritoneal cavity through the midline abdominal incision (1.5 cm). The wound was covered by sterile 37°C normal saline saturated gauze to minimize dehydration and loss of body heat. Pigs in the sham group that were not subjected to CA were used as controls. Arterial blood gas values were measured regularly using an ABL 520 Blood Gas Analyzer (Radiometer, Bronshoj, Denmark) at six time points: at baseline, 30 min, and 1, 2, 4, 6 h after ROSC. Mean aortic pressure (MAP) was monitored via the right femoral arterial catheter. The amounts of infused fluid and urine output were also monitored during the experiment. Coronary perfusion pressure (CPP) was calculated as the difference between decompression diastolic aortic and time-coincident right atrial pressure measured at the end of each minute of precordial compression. During CPR, CPP was calculated as the difference between the mean aortic and mean right atrial pressures during diastole (spontaneously beating) or decompression (CPR). CPR compression force, rate, and depth were controlled and continuously recorded during all experiments to assure that all groups received identical CPR quality.

### Experimental protocol

**Figure 1:**
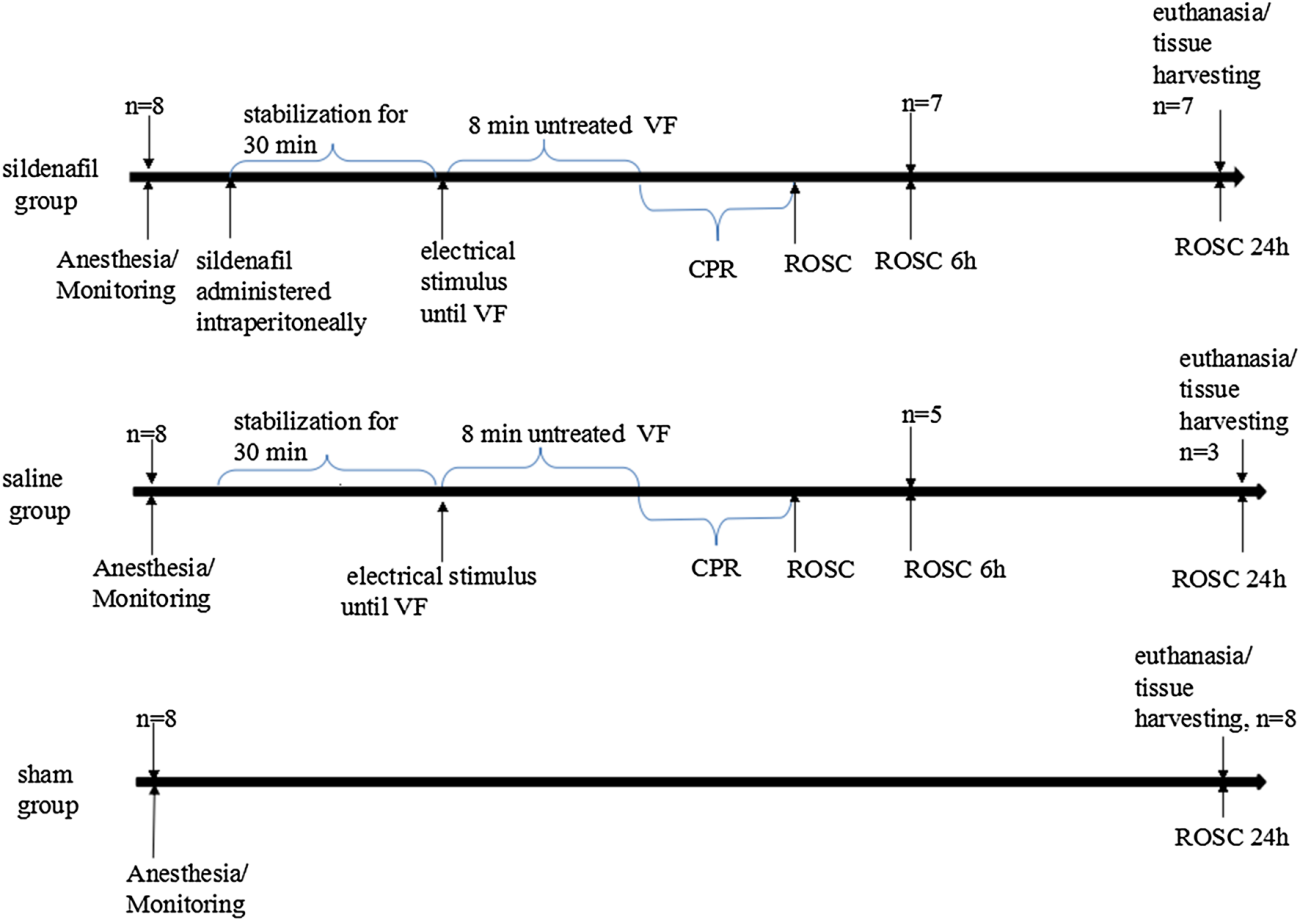
Experimental procedure. Blood samples were collected at baseline and 0.5, 1, 2, 4, and 6 h after ROSC. *CPR* cardiopulmonary resuscitation, *ROSC* restoration of spontaneous circulation, *VF* ventricular fibrillation.

The experimental procedure of CPR was performed as follow (Figure 1). After establishment of vascular catheters, the animals were allowed to equilibrate for 30 min to achieve a stable resting level. Baseline measurements and arterial blood gases were obtained. Mechanical ventilation was established as described above. The temporary pacemaker conductor was inserted into the right ventricle through the right sheathing canal and connected to an electrical stimulator (GY-600A; Kaifeng Huanan Equipment Co, Ltd, Kaifeng, China) programmed in the S_1_S_2_ mode (300/200 ms), 40 V, 8:1 proportion, and 10 ms step length to provide a continuous electrical stimulus until VF [16]. VF was defined as a waveform of VF emerging on the monitor and a rapid decline in MAP toward zero. After successful induction of VF, mechanical ventilation was discontinued. Mechanical ventilation was discontinued after the onset of VF. After 8 min of untreated VF, CPR was performed. Manual chest compressions were immediately initiated at a rate of 100 compressions per minute for 2 min and ventilation conducted using a bag respirator attached to an endotracheal tube with room air. CPR was performed by the same CPR technician from our laboratory, who compressed the porcine chest to approximately one-third of the anteroposterior diameter. The quality of chest compressions was controlled by a Heart Start MRx Monitor/Defibrillator with Q-CPR (Philips Medical Systems, Best, Holland) [17]. The compression-to-ventilation ratio was 30:2. After 2 min of CPR, a single 120 J biphasic electrical shock was attempted with a Smart Biphasic defibrillator (Philips Medical Systems, Andover, MA, USA). If the first defibrillation was unsuccessful, epinephrine (20 μg/kg) was given intravenously followed by 2 min of CPR, and repeated every 2 min if ROSC was not achieved. The 150 J shocks were used for the second and all subsequent attempts. The study was blinded as to the medication used, and only the principal investigator, who did not take part in any resuscitation effort, knew the assignment of each animal. Furthermore, the investigators involved in data recording, data entry, and data analysis were also blinded to the allocation. If spontaneous circulation was still not achieved, CPR was continued for a further 2 min, and defibrillation was attempted once more.

ROSC was defined as 10 consecutive minutes of maintenance of systolic blood pressure at 50 mmHg. If spontaneous circulation was not restored within 30 min, we regarded the animal as dead [18]. All the animals received normal saline (10 mL/kg/h) intraoperatively to replenish fluid losses. After successful resuscitation, the animals were mechanically ventilated with 100% inspired oxygen for the first 30 min, 50% for the second 30 min and 21% thereafter. With the exception of one jugular vein sheath that was used for fluid administration, all other vascular sheaths and endotracheal tube were removed after a 6 h intensive care period. The animals were allowed to recover from anesthesia, and were then placed in observation cages and monitored for a further 18 h. After a period of 24 h, post-resuscitation measurements were completed. All catheters were removed and wounds were surgically sutured. The animals were then euthanatized with 10 mL of 10 mol/L potassium chloride intravenously following a bolus of 100 mg of propofol intravenously. Myocardial specimens were harvested and snap frozen in liquid nitrogen and stored at −80°C.

### Measurements

#### Echocardiographic evaluation of left ventricular function

A transthoracic echocardiogram was obtained on all survivors at six time points: at baseline, 30 min, and at 1, 2, 4 and 6 h after ROSC. Images were obtained from the right parasternal window that provides similar views as the long and short parasternal windows in humans. Left ventricular ejection fraction (LVEF) was assessed using Simpson’s method of volumetric analysis by an independent clinical echocardiographer blinded to the treatments. Before echocardiographic evaluation, any inotropic support was stopped for at least 20 min and, if needed, was restarted immediately after the echocardiographic evaluation.

#### Western blot analysis

Myocardium sections stored at −80°C were homogenized in protein extraction solution (PRO-PREP; iNtRON, Sungnam-si, Korea). Proteins from cardiac left ventricle were prepared by rapid homogenization in Tissue Extraction Reagent II (Invitrogen Corporation, Carlsbad, CA, USA) according to the manufacturer’s instructions. The homogenates were centrifuged (14,000*g*, 15 min, 4°C), and the protein concentrations in the supernatant were determined using the Bradford method (Bio-Rad, Hercules, CA, USA). The centrifuged proteins were mixed with the same volume of 2× SDS sample buffer [62.5 mM, Tris HCl (pH 6.8), 6% (wt/vol) SDS, 30% glycerol, 125 mM DTT, and 0.3% (wt/vol) bromophenol blue], so that the ultimate concentration of the mixture was 1 × (500 μg/100 μL). The homogenates were heated at 94°C for 5 min in Laemmli sample buffer. Then, 40 μg of total protein were loaded in a stacking polyacrylamide gel and resolved on 8 and 15% polyacrylamide gels with a standard biotinylated molecular weight marker. The samples were wet-transferred to a 0.2 μm nitrocellulose membrane (Amersham Pharmacia), which was stained with Coomassie blue to assess equal loading of protein. Subsequently, the blots were blocked for 2 h with 5% nonfat dry milk in TBST buffer (10 mM Tris–HCl, pH 7.4, 100 mM NaCl, and 0.1% Tween 20) and incubated overnight at 4°C with 1:1,000 cleaved/total caspase-3 antibody, Bcl-2 (Cell Signaling Technology, Beverly, MA, USA), and 1:1,000 iNOS, eNOS, Bax, and β-actin antibody (Santa Cruz Biotechnology, Santa Cruz, CA, USA). After washing in TBST, the blots were incubated with 1:1,000 secondary anti-rabbit or anti-goat IgG-HRP-linked antibody (Cell Signaling Technology) for 1 h. The blots were washed again in TBST, and the bands were detected using enhanced chemiluminescence (Millipore, Billerica, MA, USA) and exposed to film. The optical density for quantification was obtained with Gel-Pro Analyzer version 3.1 (Media Cybernetics, Silver Spring, MD, USA). At least two membranes with duplicate sample pools were analyzed [19].

#### Quantitative real-time PCR assay

Total RNA was extracted from myocardium sections stored at −80°C using an RNeasy Mini Kit (Qiagen, Hilden, Germany). cDNA was synthesized from 1 μg of total RNA using an oligo dT primer (Amersham Pharmacia, Piscataway, NJ, USA), dNTPs (Amersham Pharmacia), Moloney murine leukemia virus reverse transcriptase (GIBCOBRL, Grand Island, NY, USA), 0.1 M DTT, and buffer in a volume of 20 μL. Then, the 20 μL of cDNA were diluted to a total volume of 100 μL. Using SYBR Green PCR Master Mix (Applied Biosystems, Foster City, CA, USA), PCR was used to amplify cDNA for iNOS [primers 5′-TCC CAG ACC CCA TAA CAA CAG-3′ (sense) and 5′-TGA GGG TGC AGC GAA CTT TA-3′ (antisense)], eNOS [5′-TCC CAG ACC CCA TAA CAA CAG-3′ (sense) and 5′-TGA GGG TGC AGCGAA CTT TA-3′ (antisense)], caspase-3, sense 5-CATGGCCTGTCAGAAAATAC-3′ (sense) and antisense 5-TAACCCGAGTAAAATGTGC-3′ (antisense); and GAPDH [5′-TCC CAG ACC CCA TAA CAA CAG-3′(sense) and 5′-TGA GGG TGC AGC GAA CTT TA-3′ (antisense)]. The amplification reaction volume was 20 μL, which consisted of 10 μL iQ SYBR Green PCR Master Mix, 1 μL primers, 1 μL cDNA, and 8 μL H_2_O. Amplification and detection were performed using a thermal cycler (Rotor-Gene 6000; Corbett Research, Mortlake, NSW, Australia). The PCR conditions consisted of denaturation at 95°C for 10 min, followed by 40 cycles consisting of 10 s at 95°C, 15 s at 60°C, and 20 s at 72°C. The fluorescence of SYBR green was measured at the end of each cycle using the comparative threshold cycle (Ct) method. The cDNA was quantified using the following formula 2^−ΔΔct^ = 2^−[(Ct of target gene − Ct of GAPDH in treated pigs − (Ct of target gene − Ct of GAPDH in sham pigs))]^.

#### cGMP measurement

One hundred mg myocardium tissues were chopped and incubated in cell lysis buffer (R&D Systems, Minneapolis, MN, USA). Then, the cells were frozen at −20°C and thawed with gentle mixing. The freeze/thaw cycle was repeated twice. The mixture was centrifuged (600*g*, 10 min, 4°C) to remove cellular debris. The supernatant was aliquoted and stored at −20°C for cGMP determination. cGMP levels were assayed in duplicate using an ELISA kit (R&D Systems, Minneapolis, MN, USA). A set of standards was assayed in duplicate with the samples. Nonspecific binding and background signals were subtracted from each reading, and the average optical density was calculated. Values are presented as picomoles of cGMP per milligram protein.

#### Micro-RNA isolation and expression

Total RNA samples were extracted using Trizol (Invitrogen, USA) from cultural myocytes. miR-1 and miR-133a level were quantified by the mirVana qRT-PCR (quantitative real-time PCR) miRNA Detection Kit (Ambion, USA) in conjunction with real-time PCR with SYBR Green I (Applied Biosystems, USA), as previously described in detail [20]. Reverse transcription primers for miR-133a was: 5′-GTCGTATCCAGTGCGTGTCGTGGAGTCGGCAATTGCACTGGATACGACCAGCTG-3′; miR-1 was: 5′-GGCTGCCGACCGTGTCGTGGAGTCGGCAATTGGTCGGCAGCCATACACAC-3. The following primers were used for PCR detection: 5′-GGGTTTGGTCCCCTTCAA-3′ (forward); 5′-AGTGCGTGTCGTGGAGTC-3′ (reverse). U6 was used as an internal control. The relative expression of miR-133a and miR-1 were calculated and normalized to U6 using the comparative Ct method. Relative expression intensity values were calculated as 2^−ΔΔCt^.

#### Detection of myocardial apoptosis

Apoptosis of cardiomyocytes and heart tissue was evaluated by terminal deoxynucleotidyl transferase-mediated dUTP nick end labeling (TUNEL) staining, which was carried out strictly according to the manufacturer’s instructions (Roche Molecular Biochemicals). Briefly, the heart tissue was embedded by paraffin and cut into sections 4–5 μm thick. The investigators were blinded to the intervention. For each specimen, cells with positive nuclei staining from five microscopic fields (400× magnification) were counted. An experienced pathologist, blinded to group assignment, counted TUNEL-positive cells under a light microscope (Shanghai Zousun Optical Instrument Co., Ltd, Shanghai, China). The apoptotic index was calculated as a ratio of the apoptotic cell number to the total cardiac myocytes number in each experimental group [21].

#### Ultra structural analysis

The remaining tissue was preserved in 10% formaldehyde and 4% paraformaldehyde to observe pathologic and ultra structural changes of the myocardium under transmission electron microscope (TEM) (H-7650; Hitachi, Tokyo, Japan). The pathologic data were assessed by reviewers blinded to the experimental groups.

#### Micro-circulation measurements

Micro-circulations were visualized at 30 min, and at 1, 2, 4 and 6 h after ROSC with the aid of a side stream dark field (SDF) imaging device (MicroScan; MicroVision Medical Inc., Amsterdam, Netherlands) with a 5× optical probe. For the measurements of intestinal microcirculation, a 2–3 cm segment of the jejunum was withdrawn with its neurovascular supply intact and cushioned with warm saline-soaked gauze [22]. The jejunal microcirculation was assessed on the anti-mesenteric aspect of the serosal side. After microcirculatory measurements were taken, the abdominal contents were then returned into the peritoneal cavity and the abdomen was closed in two layers. The muscular layer of the abdominal wall was sutured in a continuous pattern, and the skin incision was closed with wound clips. Three discrete fields were captured with precaution to minimize motion and pressure artifacts. Microvascular images were recorded on a DVD disk using a DVD recorder (Model DMREZ47V; Panasonic AVC Networks, Dalian, China). Individual images were analyzed offline. Microcirculatory flow index (MFI) was quantitated by the method of Boerma et al. [23]. The image was divided into four quadrants and the predominant type of flow (absent = 0, intermittent = 1, sluggish = 2 and normal = 3) was assessed in the small vessels of each quadrant, which were less than 20 min diameter. The MFI score represented the average values of four quadrants. Perfused vessel density (PVD) was measured based on the method of De Backer et al. [24]. Vessel density was calculated as the number of vessels crossing the lines divided by the total length of the lines. Vessel size was measured with a micrometer scale superimposed in the video display. All recordings were analyzed by three independent observers.

### Statistical analyses

Continuous variables were presented as mean ± standard deviation (SD) when data were normally distributed or as a median (25th, 75th percentiles) when data were not normally distributed. Student *t* test was used for comparisons between every two groups. Differences at different time points were compared with repeated-measures analysis of variance (ANOVA) with Bonferroni correction for post hoc comparison. In addition, the continuous variables were fixed to normal distribution and equal variances by Kolmogorov–Smirnov test and homogeneity of variance test. A value of p < 0.05 was considered as statistically significant. All analyses were conducted using the SPSS 17.0 software (SPSS Inc, Chicago III) and GraphPad PRISM version 6 (GraphPad Software Inc., San Diego, CA, USA).

## Results

A total of 24 pigs were included in the study. Eight control pigs were not subjected to CA and served as baseline, while the other 16 pigs underwent CA and CPR. There were no significant baseline differences between treatment groups in any hemodynamic parameters or respiratory parameters (Tables 1, 2) (p > 0.05).

**Table 1:** Haemodynamics and success of resuscitation

Group	Parameter	Baseline	ROSC 0 min	ROSC 30 min	ROSC 1 h	ROSC 2 h	ROSC 4 h	ROSC 6 h	Number of shocks to initial ROSC	Total adrenaline dose (mg)	6 h survival	24 h survival
Saline group	HR	101.38 ± 8.30	132.2 ± 11.3**	129.4 ± 10.4**	120.3 ± 8.9**	118.9 ± 9.3**	113.5 ± 17.4*	101.4 ± 10.4	4.9 ± 1.2	1.9 ± 0.7	5 (8)	3 (8)
	CO	2.99 ± 0.20	0.91 ± 0.07**	0.88 ± 0.06**	0.89 ± 0.06**	0.91 ± 0.05**	1.04 ± 0.05**	1.15 ± 0.04**				
	MAP	91.88 ± 3.22	125.2 ± 7.3**	122.5 ± 6.4**	115.4 ± 6.1**	109.8 ± 5.7**	107.6 ± 5.1**	101.5 ± 4.8*				
	CPP	44.2 ± 4.12	22.3 ± 2.5**	23.4 ± 2.6**	25.6 ± 2.7**	29.7 ± 3.6**	32.5 ± 3.8*	33.8 ± 3.7*				
	MPAP	24.1 ± 2.2	49.7 ± 6.3**	47.3 ± 5.7**	43.8 ± 4.7**	39.7 ± 4.2**	35.8 ± 3.7*	35.1 ± 3.1*				
	LVEF %	41.3 ± 7.4	24.3 ± 1.2**	26.4 ± 2.3**	29.4 ± 2.7**	32.3 ± 3.5*	33.5 ± 4.3*	34.6 ± 3.3*				
Sildenafil group	HR	102.18 ± 7.30	123.2 ± 9.3**^Δ^	118.4 ± 8.4**^Δ^	115.3 ± 7.9**	110.9 ± 6.3*^Δ^	107.5 ± 5.4^Δ^	105.4 ± 4.4	2.1 ± 0.6^Δ^	0.8 ± 0.3^Δ^	7 (8)	7 (8)^Δ^
	CO	2.99 ± 0.20	0.99 ± 0.06**	1.12 ± 0.03**	1.23 ± 0.04**^Δ^	1.34 ± 0.08**^Δ^	1.42 ± 0.07**^Δ^	1.36 ± 0.08**				
	MAP	89.88 ± 3.22	117.2 ± 7.1**^Δ^	115.5 ± 5.9**^Δ^	111.8 ± 5.6**	103.6 ± 4.7**^Δ^	99.8 ± 4.3*^Δ^	94.5 ± 3.4				
	CPP	43.1 ± 3.34	27.6 ± 2.8**^Δ^	28.2 ± 2.9**^Δ^	32.4 ± 3.1**	35.4 ± 3.6*^Δ^	36.5 ± 3.9^Δ^	39.4 ± 4.3^Δ^				
	MPAP	24.6 ± 2.7	40.3 ± 5.6**^Δ^	38.4 ± 4.1**^Δ^	37.8 ± 4.1**^Δ^	33.6 ± 3.5*^Δ^	29.8 ± 3.1^Δ^	28.7 ± 2.2^Δ^				
	LVEF %	43.1 ± 6.9	27.3 ± 1.3**	30.7 ± 2.8**	32.3 ± 3.1*	38.7 ± 3.9*^Δ^	39.5 ± 4.1^Δ^	40.9 ± 4.1^Δ^				

Values are mean ± SD. Pressures are given in mmHg, flows in mL/min.

*HR* heart rate, *CO* cardiac output, *MAP* mean aortic pressure, *CPP* coronary perfusion pressure, *MPAP* mean pulmonary arterial pressure, *LVEF* left ventricular ejection fraction.

* p < 0.05 vs. baseline, ** p < 0.01 vs. baseline, ^Δ^p < 0.05 vs. SA. (Student *t* test was used for comparisons between two groups. Differences at different time points were compared with repeated-measures analysis of variance (ANOVA) with Bonferroni correction for post hoc comparison).

**Table 2:** Arterial blood gasses during cardiopulmonary resuscitation and after return of spontaneous circulation

Group	Parameter	Baseline	ROSC 0 min	ROSC 30 min	ROSC 1 h	ROSC 2 h	ROSC 4 h	ROSC 6 h
Saline group	PH	7.41 ± 0.11	7.21 ± 0.05**	7.23 ± 0.07**	7.24 ± 0.06**	7.27 ± 0.09**	7.30 ± 0.08**	7.32 ± 0.09*
	PaO_2_ (mmHg)	89.1 ± 9.3	44.5 ± 4.7**	55.4 ± 5.1**	60.1 ± 6.2**	70.2 ± 7.1**	79.5 ± 7.7*	80.7 ± 7.5*
	PaCO_2_^−^ (mmHg)	39.3 ± 3.2	48.3 ± 5.1**	46.7 ± 5.4**	45.4 ± 4.1**	43.8 ± 4.7*	41.6 ± 4.2	41.5 ± 4.8
	HCO_3_^−^ (mmol/L)	27.2 ± 4.7	19.3 ± 1.2**	20.4 ± 1.6**	21.6 ± 2.7**	22.7 ± 2.6**	24.5 ± 3.1*	25.2 ± 3.7
	Lactate (mmol/L)	2.6 ± 0.5	11.2 ± 3.3**	9.3 ± 2.7**	8.8 ± 1.9**	6.9 ± 2.2**	5.8 ± 1.3*	4.1 ± 1.1
Sildenafil group	PH	7.42 ± 0.13	7.27 ± 0.07**^Δ^	7.29 ± 0.04*^Δ^	7.30 ± 0.07*^Δ^	7.32 ± 0.05*	7.35 ± 0.04*	7.38 ± 0.11
	PaO_2_ (mmHg)	90.2 ± 8.6	52.7 ± 5.3**^Δ^	63.5 ± 6.2**^Δ^	70.3 ± 7.1**^Δ^	79.3 ± 7.7*^Δ^	83.5 ± 8.1*	84.9 ± 8.2
	PaCO_2_^−^ (mmHg)	39.5 ± 3.4	44.7 ± 4.3*^Δ^	43.2 ± 4.4*^Δ^	42.4 ± 4.1*	41.3 ± 4.2	40.5 ± 4.2	41.1 ± 3.8
	HCO_3_^−^ (mmol/L)	27.6 ± 4.1	22.6 ± 2.7**^Δ^	23.7 ± 2.6**^Δ^	24.6 ± 2.7*^Δ^	25.3 ± 2.6	25.7 ± 3.1	26.3 ± 3.8
	Lactate (mmol/L)	2. 7 ± 0.4	9.5 ± 2.4**^Δ^	8.1 ± 2.1**^Δ^	7.4 ± 1.6**^Δ^	5.3 ± 1.3**^Δ^	4.2 ± 1.2*^Δ^	3.6 ± 0.7

Values are mean ± SD.

* p < 0.05 vs. baseline, ** p < 0.01 vs. baseline, ^Δ^p < 0.05 vs. saline. (Student *t* test was used for comparisons between every two groups. Partial pressures are given in mmHg. HCO_3_^−^: bicarbonate in mM; Differences at different time points were compared with repeated-measures analysis of variance (ANOVA) with Bon-ferroni correction for post hoc comparison between multiple experimental groups).

### Return of spontaneous circulation and survival

There were no significant differences in ROSC 6 h survival between the sildenafil and SA groups (Table 1). In the SA group, 6/8 animals achieved ROSC, and 5/8 animals survived 6 h. Only 3/8 animals survived 24 h. In the sildenafil group, 8/8 animal had initial ROSC and 7/8 survived to 6 and 24 h (p = 0.02 for 24 h survival rate). Animals in the sildenafil group were significantly more stable and received significantly less epinephrine than in the SA group during the recovery period (Table 1). The number of shocks to achieve ROSC was significantly lower in the sildenafil group compared to SA group 2.1 ± 0.6 vs. 4.9 ± 1.2, respectively.

### Hemodynamics and left ventricular function

Each pig developed a marked post-resuscitation myocardial dysfunction during the 6 h of observation. Indeed, HR and MAP were significantly increased during the first 4 h after ROSC compared to baseline (p < 0.01 vs. baseline, Table 1). However, sildenafil pretreatment inhibited this increase of HR and MAP at four time-points (0 min, 30 min, 2h and 4 h after ROSC), and CPP and MPAP remained increased during the initial 6 h after ROSC when compared to baseline values (p < 0.01) and, and the values of CPP and MPAP in the sildenafil group were continuously lower than those in the SA group at most time points (p < 0.05, respectively). While CO was decreased during the initial 6 h after ROSC when compared to baseline values (p < 0.01) and the CO values were significantly higher in the sildenafil group than those in the SA group at 1, 2 and 4 h after ROSC (p = 0.03, p = 0.02, p = 0.04, respectively (Table 1).

Echocardiographic evaluation revealed that animals receiving saline alone had a significantly lower LVEF than the animals treated with sildenafil at 2 h after ROSC (32.3 ± 3.5, vs 38.7 ± 3.9%, p < 0.01). The effect was maintained at 6 h after ROSC (34.6 ± 3.3 vs. 40.9 ± 4.1%, p < 0.01, Table 1).

### Arterial blood gases

There were no significant differences in blood gas values at baseline among three groups. PH, HCO_3_^−^ and lactate were significantly lower in the sildenafil group than those in the SA group at 0 min, 30 min and 1 h after ROSC (p = 0.04, p = 0.02, p = 0.02 respectively) and the PaO_2_ was higher in the sildenafil group than those in the SA group at 0 min, 30 min, 1 h and 2 h after ROSC (p = 0.03, p = 0.04, p = 0.02, p = 0.03, respectively, Table 2).

### Effect of sildenafil on expression of Bax and Bcl-2 in myocytes: role of NO signaling

**Figure 2:**
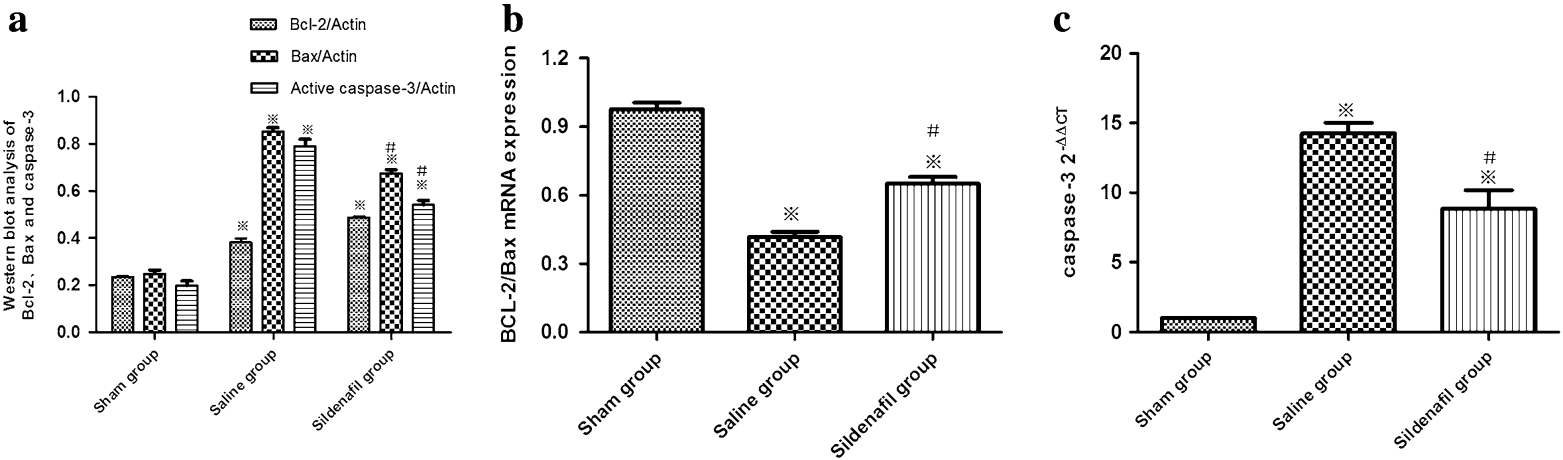
**a** Western blots of quantification of Bax, Bcl-2 and active caspase-3 protein levels of myocardial tissue in the sham, saline and sildenafil groups at 24 h after ROSC. **b** Expressions of Bcl-2/Bax proteins of myocardial tissue at 24 h after ROSC. **c** Quantification of mRNA expressions of caspase 3. The *value* represent mean ± SD. *p < 0.05, **p < 0.01 vs. sham, ^#^p < 0.05 vs. saline (one-way repeated-measures ANOVA).

The balance of Bax and Bcl-2 plays an important role in the process of apoptosis, we therefore examined the effect of sildenafil on Bax and Bcl-2 expression. The level of Bax was elevated in the myocardium of SA group compared with the myocardiums of the sham group (p < 0.05). Pretreatment with sildenafil prevented the elevation of Bax in the myocardiums of I/R pigs. No difference was observed in Bcl-2 levels between sildenafil and SA groups. The ratio of Bax to Bcl-2 is calculated as an index of apoptotic signaling. Bcl-2/Bax was decreased in myocardium and this decrease was significantly reversed by treatment with sildenafil, suggesting that sildenafil exerted the anti-apoptotic effect by up-regulation Bcl-2/Bax radio than vehicle-treated pigs (p < 0.05, Figure 2a, b).

### Caspase-3 activity assay

Compared with sham group, the caspase-3 activity was increased significantly in saline group and sildenafil group (p < 0.05), and compared with the saline group, sildenafil administration reduced the caspase-3 activity (p < 0.05, Figure 2c).

### TUNEL assay of cardiomyocyte apoptosis

**Figure 3:**
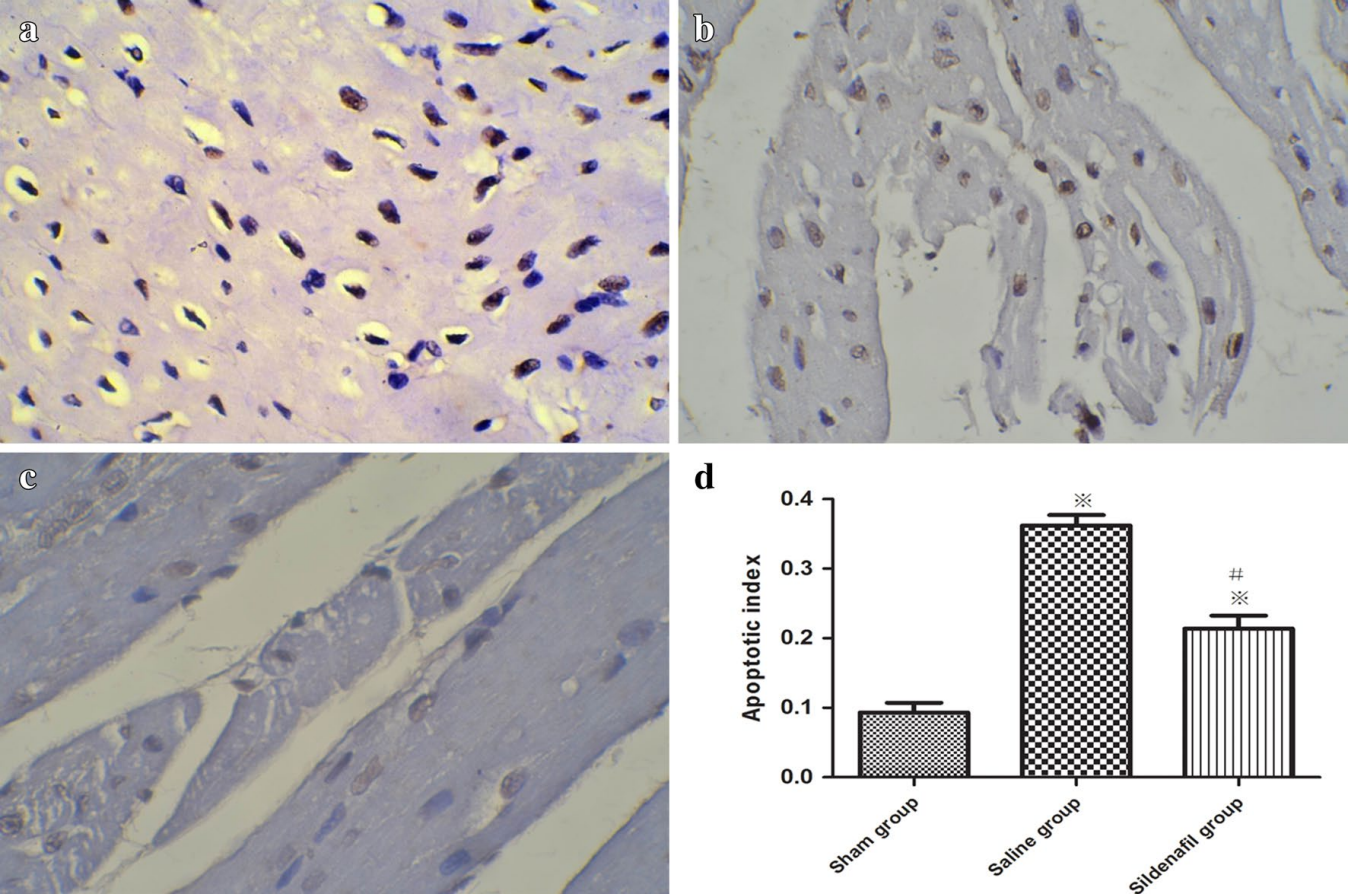
TUNEL analysis of cardiomyocyte apoptosis in the sham (**a**), saline (**b**) and sildenafil (**c**) groups at 24 h after ROSC: the *brown nuclei* indicate TUNEL-positive nuclei. Magnification, ×400. **d** Apoptosis index. The *value* represent mean ± SD. *p < 0.05, **p < 0.01 vs. sham, ^#^p < 0.05 vs. saline.

As shown in Figure 3a–c, the brown nuclei indicate TUNEL-positive nuclei. TUNEL-positive cells were recognized in affected myocytic nuclei with chromatin condensation in both core and marginal zones. These cells were more common in the SA group, which were distributed across the lesion, whereas, there were fewer TUNEL-positive cells in the sildenafil group and the cells that were present were thinly scattered in the lesion. Figure 3d shows that, at 24 h after ROSC, the amounts of TUNEL positive cardiomyocytes was increased in the SA group compared with sham group, but this increase was alleviated by sildenafil (p < 0.05).

### Expression of cGMP and eNOS/iNOS

**Figure 4:**
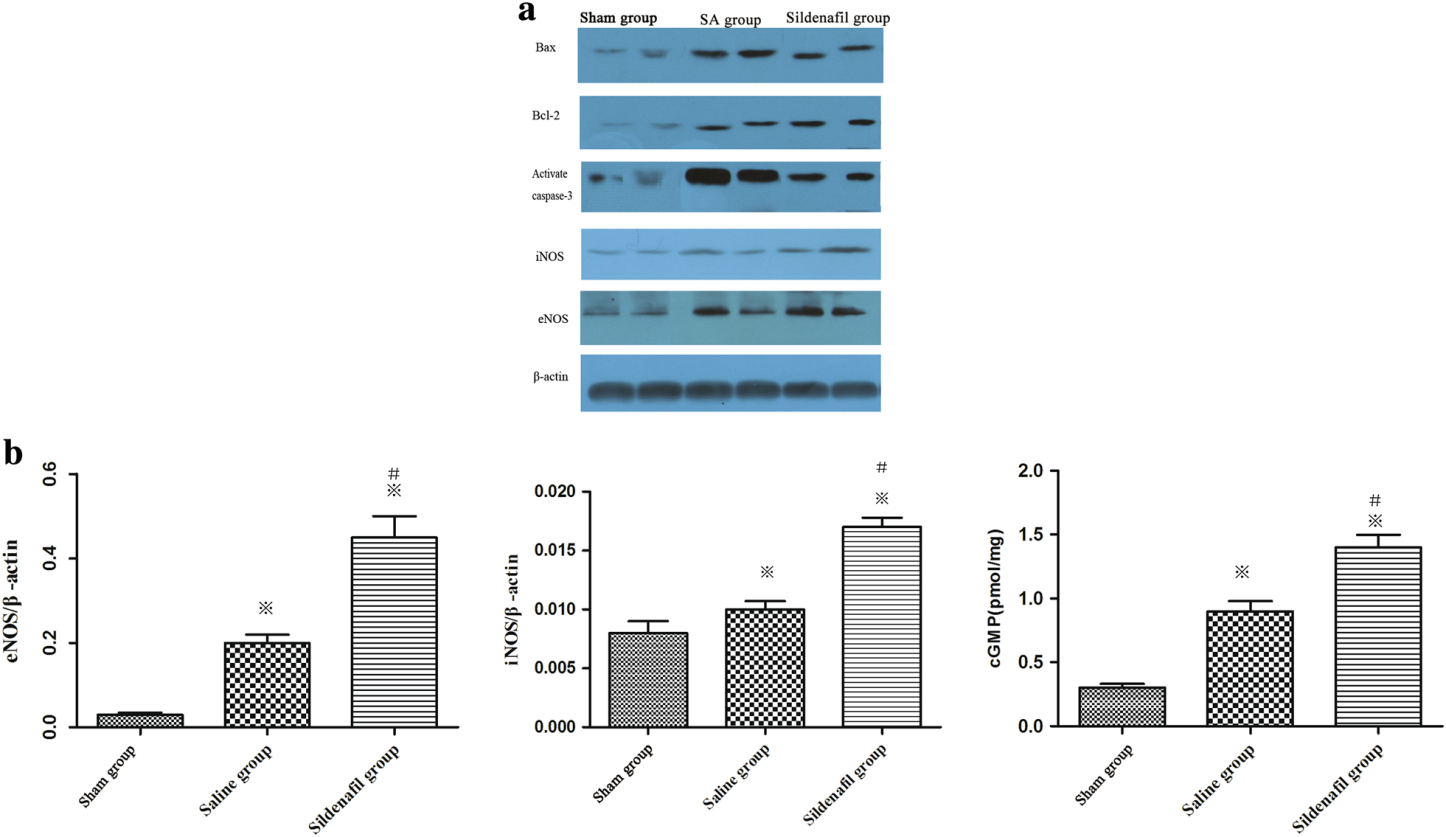
Western blot of **a** expression of Bax, Bcl-2 and active caspase-3, cGMP, eNOS and iNOS proteins of myocardial tissue in the sham, SA and sildenafil groups at 24 h after ROSC. **b** Quantification of cGMP, eNOS and iNOS accumulation protein levels. The *value* represent mean ± SD. *p < 0.05, **p < 0.01 vs. sham, ^#^p < 0.05 vs. saline.

cGMP and eNOS/iNOS accumulation were measured in heart samples at 24 h after ROSC in sham-operated, sildenafil-pretreated and vehicle-treated pigs. cGMP and eNOS/iNOS accumulation increased significantly in the sildenafil-treated group, compared with the vehicle treated group and sham-operated group at 24 h after ROSC. Furthermore, significantly greater protein levels of cGMP, eNOS and iNOS in the myocardiums were achieved in sildenafil group when compared with SA group 24 h after ROSC (p < 0.05, Figure 4).

### Changes in miRNA-1 and miRNA-133 expression

**Figure 5:**
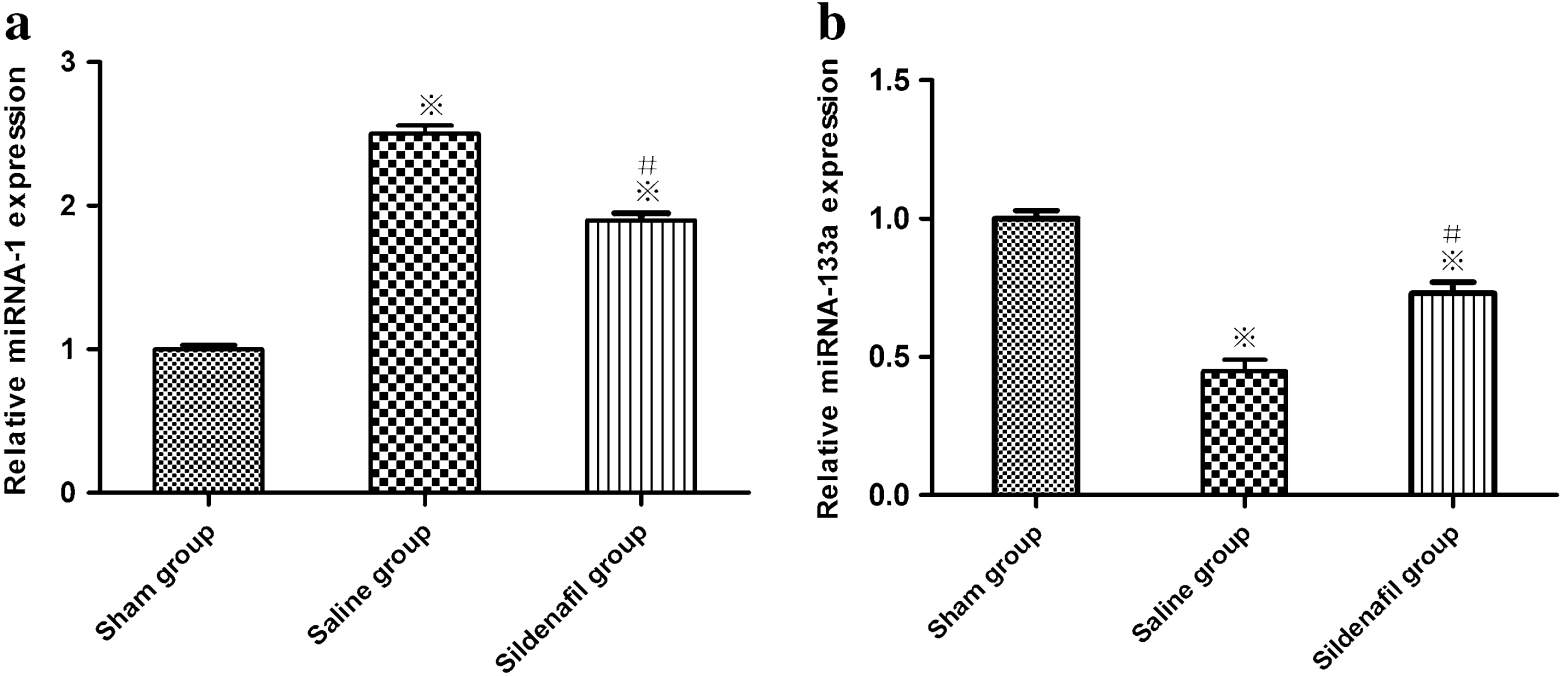
Real-time PCR analysis of relative microRNA-1(**a**) and microRNA-133a (**b**) expression. The *value* represent mean ± SD. *p < 0.05, **p < 0.01 vs. sham, ^#^p < 0.05 vs. saline.

Real-time PCR analysis showed that miR-1 expression was significantly up-regulated, whereas miR-133a expression was significantly down-regulated in saline group compared with the sham group, and these effects were attenuated by sildenafil (p < 0.05, Figure 5).

### Ultra structural changes in cardiomyocytes

**Figure 6:**
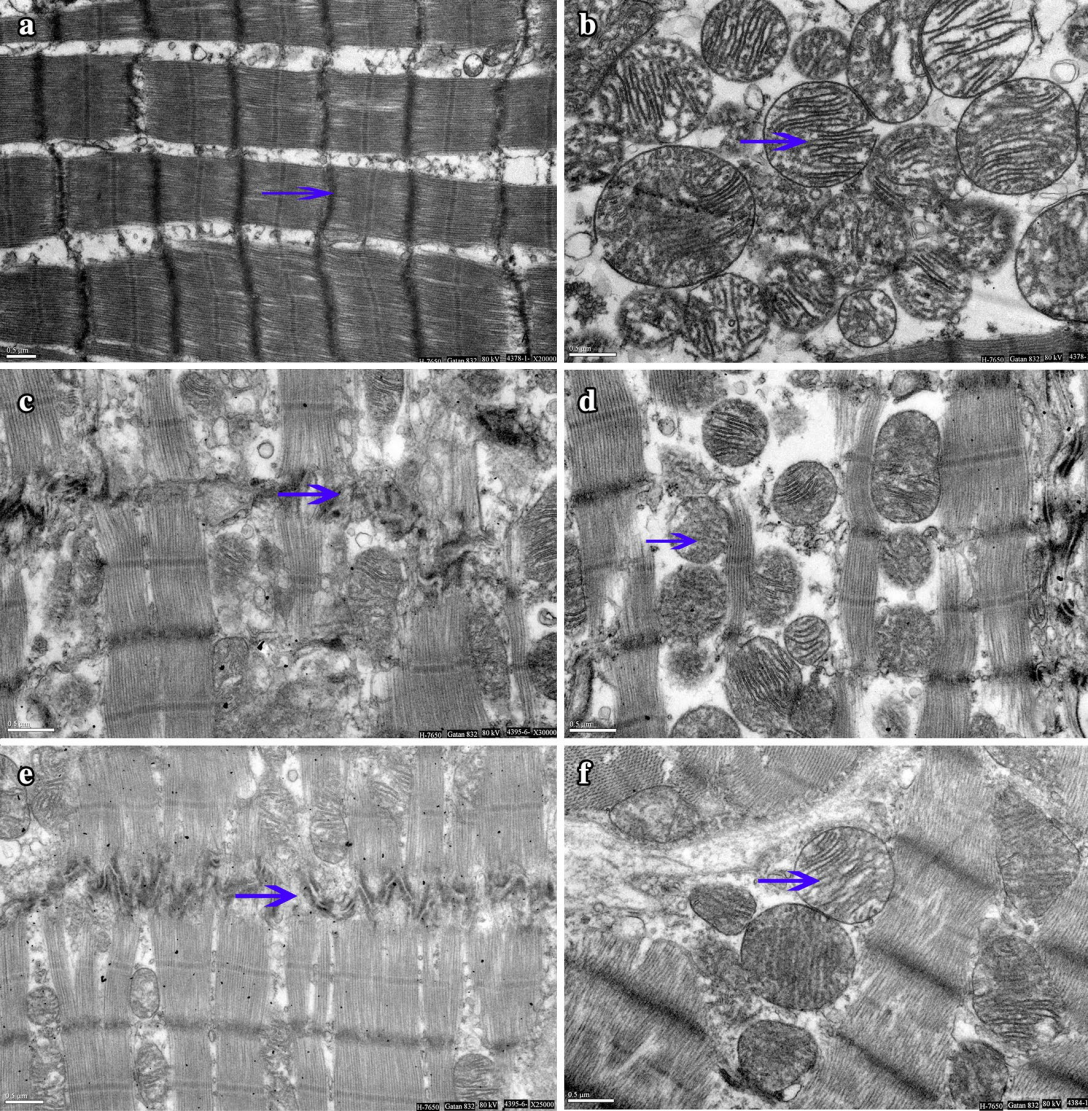
Cytoplasmic ultrastructure of the myocardium under an electron microscope: **a**, **b** normal mitochondria structures were displayed, *Z-line*, *M-line* and intercalated disk is clear in the sham group (*arrows*). **c**, **d** The myocardial fiber and intercalated disk were obviously disordered, broken, even dissolved in the SA group 24 h after ROSC (*arrows*). **e**, **f** Animals treated with sildenafil exhibited little intracellular damage in the myocardium at 24 h after ROSC (*arrows*).

Under TEM, normal mitochondria structures were displayed in the sham group (Figure 6a, b). The myocardial fiber and intercalated disk were obviously disordered, broken, even dissolved in the SA group 24 h after ROSC; most of the mitochondria were severely broken, even exhibiting vascular with vague, arranged irregularly, or disrupted cristae (Figure 6c, d). Animals treated with sildenafil exhibited little intracellular damage in the myocardium: partial nuclear chromatin condensation, reduced crest fracture and moderate edema occurred in the mitochondria and sarcoplasmic reticula (Figure 6e, f).

### Effect of the sildenafil on microcirculatory at 6 h after ROSC

**Figure 7:**
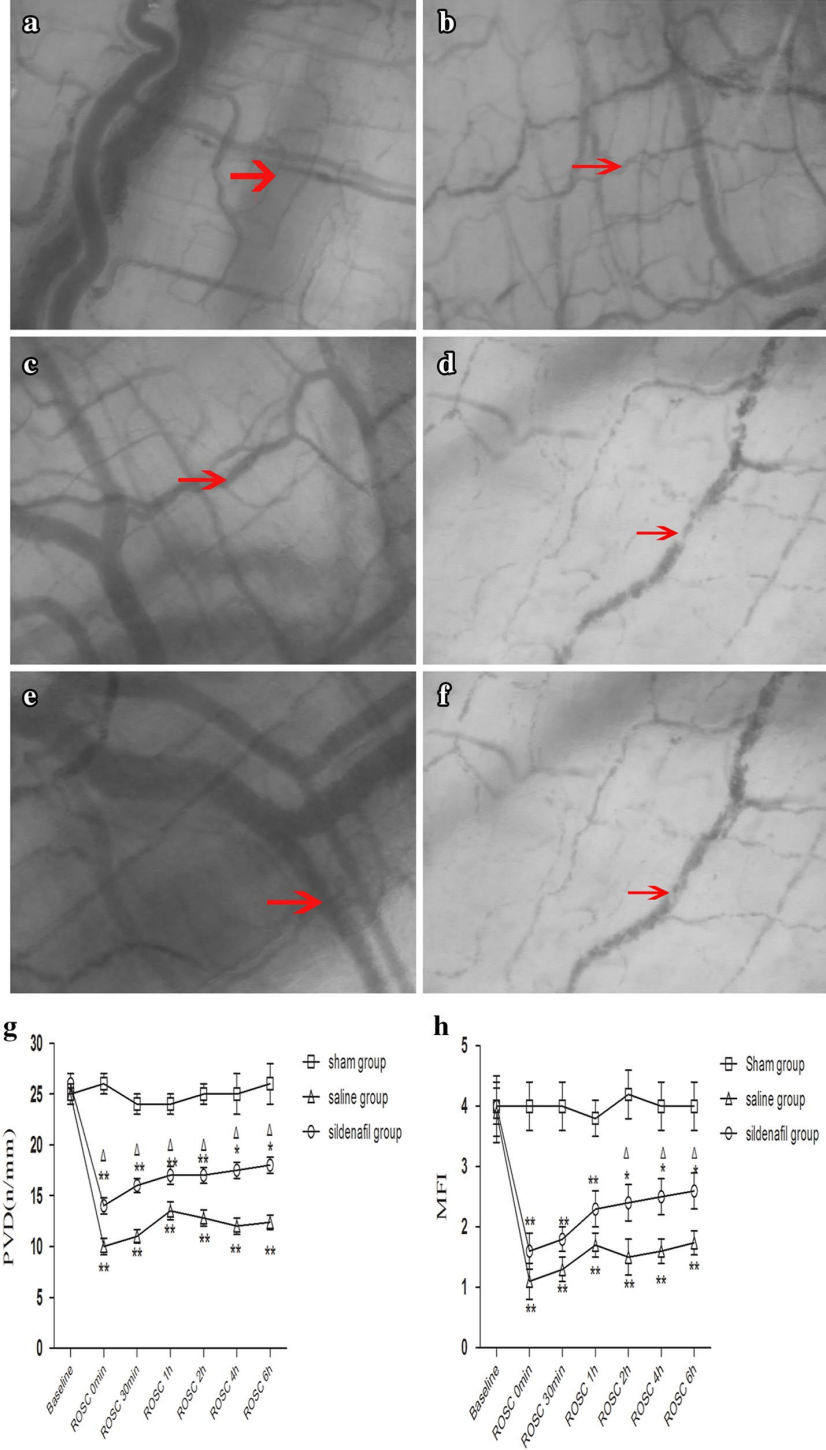
Images of the images of intestinal microcirculation, as obtained by the SDF video microscope at 6 h after ROSC in the sham (**a**, **b**) saline (**c**, **d**) and sildenafil (**e**, **f**) groups; changes of PVD (**g**) and MFI (**h**) of intestinal microcirculation in the sham, saline and sildenafil groups at 6 h after ROSC. The *values* are reported as mean ± SD. *ROSC* restoration of spontaneous circulation. *p < 0.05, **p < 0.01 vs. sham, ^Δ^p < 0.05 vs. saline.

Examples of the images of intestinal micro-vascular, as obtained by the SDF video microscope at 6 h after ROSC, are shown in Figure 7. Normal intestinal micro-vascular structures were displayed in the sham group (Figure 7a, b). Intestinal PVD was significantly reduced from 21.2 ± 1.5/mm at baseline to 12.4 ± 0.7/mm and MFI was significantly reduced from 3.0 ± 0.0 at baseline to 1.9 ± 0.2 at 6 h after ROSC in the saline group (both p < 0.05, Figure 7c, d). Contrary to a progressive recovery of both measurements observed in the sildenafil group, the significant reduction in both PVD and MFI persisted after resuscitation in the saline group (Figure 7e, f). At 6 h after ROSC, measurement of PVD and MFI were significantly lower in the saline group than in the sildenafil group: intestinal PVD (12.4 ± 0.7 vs. 16.9 ± 0.8/mm, p < 0.05, Figure 7g); intestinal MFI (1.7 ± 0.2 vs. 2.5 ± 0.5, p < 0.05, Figure 7h).

## Discussion

The major findings of this study were as follows: (1) sildenafil has protective effects against post-resuscitation myocardial dysfunction by attenuating apoptosis, as evidenced by the finding that pretreatment with sildenafil increased the expressions of iNOS/eNOS and decreased the activation of caspase-3, TUNEL positive cells, and the Bax/Bcl-2 ratio. (2) Sildenafil treatment inhibits the increases in the miR-1 levels which causes down-regulation of antiapoptotic gene, however alleviates the decreases in the miR-133a levels which negatively regulates pro-apoptotic genes. (3) Pretreatment of the sildenafil prior to VF reduces the severity of microcirculatory dysfunction during the no-flow or low-flow state of CA.

Apoptosis is one of the important mechanisms of cell death in isolated myocardiums following I/R injury. Apoptosis is associated with the up-regulation of proapoptotic protein (Bax), down-regulation of antiapoptotic protein (Bcl-2), and increased caspase-3 activity [25]. Constitutive expression of high levels of Bcl-2 protein enhances survival of many kinds’ cells including cardiomyocytes on exposure to various adverse stimuli. The Bax gene encodes a protein that is primarily localized to the cytosol and is translocated to the mitochondria after apoptotic stimulation. Homodimers of Bcl-2 or Bax associates with the mitochondrial membrane and affects membrane permeability. Down-regulation of Bcl-2/Bax might result in the activation of the caspase family of proteases, such as caspase-3, which is responsible for the induction of apoptotic cell death, leading to internucleosomal DNA fragmentation [26]. Since apoptosis represents an active, gene-directed mechanism, and is an important means with which to maintain cardiomyocyte homeostasis, it should be possible to control this process for therapeutic purposes. In the present study, we observed a significant increase in Bcl-2 and decreases in Bax and caspase-3 in the sildenafil-treated pigs compared with the vehicle-treated pigs, demonstrating the antiapoptotic capacity of sildenafil in heart I/R injury. Furthermore, the number of TUNEL-positive cells was significantly lower in the myocardiums from sildenafil-treated I/R pigs compared with the vehicle-treated I/R pigs. Our data are also consistent with a recent work demonstrating that sildenafil treatment could decrease TUNEL-positive cells in isolated adult cardiomyocytes in culture by increasing protein kinase G activity, as well as in ischemic cardiomyopathy in mice [27]. These results established a key link between NO signaling and the expression of cytoprotective antiapoptotic protein, Bcl-2. Studies in cardiomyocytes also suggested a role for NO in induction and regulation of Bcl-2. In addition, the mitochondrial ATP-sensitive potassium channel opening property of sildenafil may also contribute to its role in the inhibition of apoptosis.

MicroRNAs are an evolutionally conserved class of small regulatory RNAs that have been recently characterized as important regulators in cardiac developmental and pathological processes [28]. Furthermore, studies on specific miRNAs in animal models have identified distinct roles for miRNAs both during heart development and under pathological conditions, including the regulation of key factors important for cardiogenesis, the hypertrophic growth response, and cardiac conductance [29]. Cumulatively, these findings indicate clearly that miRNAs are important regulators of gene expression in heart development, function, and pathology. Increasing evidence indicates that miRNAs silence genes by multiple mechanisms including degrading their target mRNA [30]. Among the known miRNAs, miR-1 and miR-133 are clustered on the same chromosomal locus and transcribed together as a single transcript, which becomes two independent, mature miRNAs with distinct biological functions. miRNA-1 expression is restricted to heart and skeletal muscle and is regulated by transcription factor [31]. Indeed, miRNA-1 is one of the most strongly up-regulated miRNAs, whereas miRNA-133a is one of the down-regulated miRNAs in rat hearts upon acute myocardial I/R. Recent functional studies indicate that miRNA-1 and miRNA-133a have opposite effects in the regulation of stress-induced myocyte survival, with a pro-apoptotic role of miR-1 and anti-apoptotic role of miRNA-133a. The opposite consequences of miRNA-1/miRNA-133a may be largely attributed to different targets. Increased miRNA-1 causes down-regulation of multiple antiapoptotic genes, such as Hsp70, IGF-1 and Bcl-2; whereas miR-133a negatively regulates a pro-apoptotic gene (i.e. caspase-9) [10]. In addition, miRNA-133a has been reported to exhibit an anti-apoptotic effect in I/R by decreasing the expression of the apoptosis-related gene caspase, somehow implying protective effects of miRNA-133a on I/R-triggered cardiac remodeling [10]. Collectively, these data suggest that approaches to either decrease cardiac miR-1 levels or increase miRNA-133 levels during an ischaemic event might potentially attenuate I/R-induced myocardial injury. The results of the present study suggested that sildenafil treatment inhibited the increases in the miRNA-1 levels which causes down-regulation of antiapoptotic gene-Bcl-2, whereas alleviated the decreases in the miRNA-133a levels which negatively regulates pro-apoptotic genes. All these findings further suggest a critical role of the sildenafil in post-resuscitation myocardial dysfunction by regulating miRNAs expression. It is of interest to investigate the detailed mechanisms by which sildenafil regulates the miRNAs expression in the future.

Also, our findings indicate a possible contribution of oxidative stress to post-cardiac arrest myocardial injury and dysfunction. After I/R, endothelial dysfunction and inhibition of NOS with reduced NO availability are commonly observed, due to oxidative stress [32]. NO is an endothelium-derived vasoactive factor produced by NOS, which plays important roles in modulating coronary vascular tone and tissue perfusion [33]. Kern et al. had reported that the protein expression of myocardial NOS increases after 10 min of untreated VF, decreases somewhat during the early postresuscitation period, and then steadily increases up to 6 h postresuscitation in swine [34]. Sildenafil, a PDE-5 inhibitor, prevents the breakdown of NO-driven cGMP, primarily in vascular smooth muscle cells, and is therefore a potent vasodilator. PDE5 hydrolyses cGMP that is formed from guanosine triphosphate by NO activation of guanylate cyclases. Evidence indicates that NO through the NO-NOS/GC/cGMP (nitric oxide/nitric oxide synthase-guanyly cyclase-cyclic guanosine mono phosphate) pathway has an important function in the development of myocardial dysfunction with I/R injury [35]. The protective mechanisms of sildenafil appear to be due to activation of the NO/cGMP signaling pathway via the inhibition of PDE5 activity and coordinated induction of NOS isoform expression. The direct inhibition of PDE-5 by sildenafil may result in a higher accumulation of cGMP in the heart tissue, which has been experimentally confirmed in sildenafil-treated rat myocardium [36]. Subsequently, cGMP may activate protein kinase G that in turn opens the KATP channel, resulting in the cardioprotective effects as reported earlier [36]. These results are consistent with our findings in which the protein expression of cGMP and eNOS/iNOS were increased significantly at 24 h after ROSC in the sildenafil group compared with the saline group. Our results provided the evidence that NO was necessary in triggering the preconditioning effect in the cardiomyocytes and the resulting cardioprotection by sildenafil.

Prolonged ischemia with subsequent reperfusion may cause dysfunction of microcirculation, principally at the capillary level [37]. Re-establishment of epicardial myocardial blood flow may not immediately result in normal myocyte perfusion. A number of mechanisms for this “capillary no-reflow” phenomenon have been suggested, including intravascular microthrombiformation, leukocyte adhesion and microcirculation “plugging”, endothelial cell swelling, vasomotor dysfunction, and interstitial edema [38]. However, microcirculatory changes can be dispatched from changes in regional blood flow and differ among visceral organs especially in low-flow states. Another finding of this study was that we revealed intestinal microcirculatory changes in the early stage following resuscitation. In the present study, intestinal microcirculatory dysfunction was improved in the sildenafil group when compared to the SA group, which suggesting that sildenafil improve post-resuscitation intestinal microcirculation function and strongly supporting the pharmacological vasodilation and collateral recruitment/patency through the NO-cGMP pathway.

To interpret the results of our experimental study, it is necessary to take several limitations into consideration. First, the healthy porcine model does not always indicate the real condition of patients in a clinical setting [39]. Second, Although our porcine model is the closest before clinical trials, a one-to-one translation into clinical practice may be hampered by species-and organ-dependent differences in sensitivity to I/R injury [40]. Third, this experiment did not serially measure myocardial tissue injury biomarkers from 30 min to 24 h after ROSC. Forth, based on previous animal research and pharmacokinetics of sildenafil, the drugs were administered as a pre-treatment 30 min before VF which cannot reflect its clinical applicability truly. Fifth, for our next project, we plan to investigate the relationship between apoptosis and other possible signals mediating the effect of sildenafil on post-resuscitation myocardial dysfunction. Finally, we have not evaluated the effect of sildenafil on neurological function, however, ongoing studies will address this question.

## Conclusions

The present study demonstrated that (1) the administration of sildenafil improves the success of initial resuscitation. Our data, based on the pharmacological inhibition of NOS, also suggest an important role of NO signaling in protective effect of sildenafil against apoptosis. Additionally, sildenafil treatment inhibited the increases in the microR-1 levels which causes down-regulation of antiapoptotic gene-Bcl-2, whereas alleviated the decreases in the microR-133a levels which negatively regulates pro-apoptotic genes. (2) Pretreatment of the sildenafil prior to VF reduces the severity of microcirculatory dysfunction during the no-flow or low-flow state of CA. Overall, these results provide an important mechanistic basis of sildenafil as a potentially novel pharmacologic adjunct to resuscitation from CA for the purpose of attenuating the organ injury caused by I/R injury.

## Abbreviations

CA: cardiac arrest
cGMP: cyclic guanosine monophosphate
CO: cardiac output
CPP: coronary perfusion pressure
CPR: cardiopulmonary resuscitation
EF: ejection fraction
HR: heart rate
I/R: ischemia/reperfusion
MAP: mean aortic pressure
MPAP: mean pulmonary arterial pressure
NO: nitrogen oxide
PDE-5: isoform 5 of the enzyme phosphodiesterase
ROSC: return of spontaneous circulation
SA: saline
SHAM: sham
VO_2_: oxygen consumption

## References

[1] Nichol G, Thomas E, Callaway CW, Hedges J, Powell JL (2008). Regional variation in out-of-hospital cardiac arrest incidence and outcome. JAMA.

[2] Neumar RW, Nolan JP, Adrie C, Aibiki M, Berg RA (2008). Post-cardiac arrest syndrome epidemiology, pathophysiology, treatment, and prognostication. A consensus statement from the International Liaison Committee on Resuscitation (American Heart Association, Australian and New Zealand Council on Resuscitation, European Resuscitation Council, Heart and Stroke Foundation of Canada, Inter-American Heart Foundation, Resuscitation Council of Asia, and the Resuscitation Council of Southern Africa); the American Heart Association Emergency Cardiovascular Care Committee; the Council on Cardiovascular Surgery and Anesthesia; the Council on Cardiopulmonary, Perioperative, and Critical Care; the Council on Clinical Cardiology; and the Stroke Council. Circulation.

[3] DE Choi, Jeong JY, Lim BJ, Chung S, Chang YK, Lee SJ (2009). Pretreatment of sildenafil attenuates ischemia-reperfusion renal injury in rats. Am J Physiol Renal Physiol.

[4] Loganathan S, Radovits T, Hirschberg K, Korkmaz S, Barnucz E, Karck M (2008). Effects of selective phosphodiesterase-5-inhibition on myocardial contractility and reperfusion injury after heart transplantation. Transplantation.

[5] Zhang Q, Yuan W, Wang G, Wu J, Wang M, Li C (2014). The protective effects of phosphodiesterase-5 inhibitor, sildenafil on post-resuscitation cardiac dysfunction of cardiac arrest: metabolic evidence from microdialysis. Crit Care.

[6] Kolettis TM, Kontaras K, Spartinos I, Maniotis C, Varnavas V, Koutouzis M (2010). Dose-dependent effects of sildenafil on post-ischaemic left ventricular function in the rat isolated heart. J Pharm Pharmacol.

[7] Tili E, Michaille JJ, Gandhi V, Plunkett W, Sampath D, Calin GA (2007). miRNAs and their potential for use against cancer and other diseases. Future Oncol.

[8] Mishra PK, Tyagi N, Kumar M (2009). MicroRNAs as therapeutic target for cardiovascular diseases. J Cell Mol Med.

[9] Boštjančič E, Zidar N, Štajer D, Glavač D (2010). MicroRNAs miR-1, miR-133a, miR-133b and miR-208 are dysregulated in human myocardial infarction. Cardiology.

[10] Xu C, Lu Y, Pan Z, Chu W, Luo X, Lin H (2007). The muscle-specific miemRNAs miRNA-1 and miRNA-133 produce opposing effects on apoptosis by targeting HSP60, HSPT0 and Caspase-9 in cardiomyocytes. J Cell Sci.

[11] Thum T, Condorelli G (2015). Long noncoding RNAs and microRNAs in cardiovascular pathophysiology. Circ Res.

[12] Berg RA, Sanders AB, Kern KB, Hilwig RW, Heidenreich JW, Porter ME (2001). Adverse hemodynamic effects of interrupting chest compressions for rescue breathing during cardiopulmonary resuscitation for ventricular fibrillation cardiac arrest. Circulation.

[13] Fries Michael, Tang Wanchun, Chang Yun-Te, Wang Jinglan, Castillo Carlos, Weil MH (2006). Microvascular blood flow during cardiopulmonary resuscitation is predictive of outcome. Resuscitation.

[14] World Medical Association (2013). World Medical Association declaration of Helsinki: ethical principles for medical research involving human subjects. JAMA.

[15] Huang Q, Xu H, Yu Z, Gao P, Liu S (2010). Inbred Chinese Wuzhishan (WZS) minipig model for soybean glycinin and β-conglycinin allergy. J Agric Food Chem.

[16] Zhang Q, Li C (2013). Combination of epinephrine with esmolol attenuates post-resuscitation myocardial dysfunction in a porcine model of cardiac arrest. PLoS One.

[17] Wang S, Li CS, Ji XF (2010). Effect of continuous compressions and 30:2 cardiopulmonary resuscitation on global ventilation/perfusion values during resuscitation in a porcine model. Crit Care Med.

[18] Valenzuela TD, Roe DJ, Cretin S (1997). Estimating effectiveness of cardiac arrest interventions. Circulation.

[19] Olivetti G, Abbi R, Quaini F, Kajstura J, Cheng W (1997). Apoptosis in the failing human heart. N Engl J Med.

[20] Takagi H, Hsu CP, Kajimoto K, Shao D, Yang Y, Maejima Y (2010). Activation of PKN mediates survival of cardiac myocytes in the heart during ischemia/reperfusion. Circ Res.

[21] Gong P, Hua R, Zhang Y, Zhao H, Tang Z, Mei X (2013). Hypothermia-induced neuroprotection is associated with reduced mitochondrial membrane permeability in a swine model of cardiac arrest. J Cereb Blood Flow Metab.

[22] Qian J, Yang Z, Cahoon J, Xu J, Zhu C, Yang M (2014). Post-resuscitation intestinal microcirculation: its relationship with sublingual microcirculation and the severity of post-resuscitation syndrome. Resuscitation.

[23] Boerma EC, Mathura KR, van der Voort PH, Spronk PE, Ince C (2005). Quantifying bedside-derived imaging of microcirculatory abnormalities in septic patients: a prospective validation study. Crit Care.

[24] De Backer D, Creteur J, Preiser JC, Dubois MJ, Vincent JL (2002). Microvascular blood flow is altered in patients with sepsis. Am J Respir Crit Care Med.

[25] Geng Y, Walls KC, Ghosh AP, Akhtar RS, Klocke BJ, Roth KA (2010). Cytoplasmic p53 and activated Bax regulate p53-dependent, transcription-independent neural precursor cell apoptosis. J Histochem Cytochem.

[26] Saikumar P, Dong Z, Weinberg JM, Venkatachalam MA (1998). Mechanisms of cell death in hypoxia/reoxygenation injury. Oncogene.

[27] Elrod JW, Greer JJ, Lefer DJ (2007). Sildenafil-mediated acute cardioprotection is independent of the NO/cGMP pathway. Am J Physiol Heart Circ Physiol.

[28] Pernaute B, Spruce T, Smith KM, Sánchez-Nieto JM, Manzanares M, Cobb B (2014). MicroRNAs control the apoptotic threshold in primed pluripotent stem cells through regulation of BIM. Genes Dev.

[29] Uchida S, Dimmeler S (2015). Long noncoding RNAs in cardiovascular diseases. Circ Res.

[30] Chen JS, Wu DT (2013). Application of intronic microRNA agents in cosmetics. Methods Mol Biol.

[31] Ikeda S, He A, Kong SW, Lu J, Bejar R, Bodyak N (2009). MicroRNA-1 negatively regulates expression of the hypertrophy-associated calmodulin and Mef2a genes. Mol Cell Biol.

[32] Witting PK, Rayner BS, Wu BJ, Ellis NA, Stocker R (2007). Hydrogen peroxide promotes endothelial dysfunction by stimulating multiple sources of superoxide anion radical production and decreasing nitric oxide bioavailability. Cell Physiol Biochem.

[33] Schulz R, Smith JA, Lewis MJ, Moncada S (1991). Nitric oxide synthase in cultured endocardial cells of the pig. Br J Pharmacol.

[34] Kern KB, Berg RA, Hilwig RW, Larson DF, Gaballa MA (2008). Myocardial cytokine IL-8 and nitric oxide synthase activity during and after resuscitation: preliminary observations in regards to post-resuscitation myocardial dysfunction. Resuscitation.

[35] Bolli R (2001). Cardio-protective function of inducible nitric oxide synthase and role of nitric oxide in myocardial ischemia and preconditioning: an overview of a decade of research. J Mol Cell Cardiol.

[36] Koneru S, Varma Penumathsa S, Thirunavukkarasu M, Vidavalur R, Zhan L, Singal PK (2008). Sildenafil-mediated neovascularization and protection against myocardial ischaemia reperfusion injury in rats: role of VEGF/angiopoietin-1. J Cell Mol Med.

[37] Menger MD, Rucker M, Vollmar B (1997). Capillary dysfunction in striated muscle ischemia/reperfusion: on mechanisms of capillary no-reflow. Shock.

[38] Dauber IM, VanBenthuysen KM, McMurtry IF, Wheeler GS, Lesnefsky EJ, Horwitz LD (1990). Functional coronary microvascular injury evident as increased permeability due to brief ischemia and reperfusion. Circ Res.

[39] Riess ML, Stowe DF, Warltier DC (2004). Cardiac pharmacological preconditioning with volatile anesthetics: from bench to bedside. Am J Physiol Heart Circ Physiol.

[40] Shen YT, Vatner SF (1996). Differences in myocardial stunning following coronary artery occlusion in conscious dogs, pigs, and baboons. Am J Physiol.

